# Members of *Marinobacter* and *Arcobacter* Influence System Biogeochemistry During Early Production of Hydraulically Fractured Natural Gas Wells in the Appalachian Basin

**DOI:** 10.3389/fmicb.2018.02646

**Published:** 2018-11-15

**Authors:** Morgan V. Evans, Jenny Panescu, Andrea J. Hanson, Susan A. Welch, Julia M. Sheets, Nicholas Nastasi, Rebecca A. Daly, David R. Cole, Thomas H. Darrah, Michael J. Wilkins, Kelly C. Wrighton, Paula J. Mouser

**Affiliations:** ^1^Department of Civil, Environmental, and Geodetic Engineering, The Ohio State University, Columbus, OH, United States; ^2^Department of Civil and Environmental Engineering, Colorado State University, Fort Collins, CO, United States; ^3^School of Earth Sciences, The Ohio State University, Columbus, OH, United States; ^4^Department of Microbiology, The Ohio State University, Columbus, OH, United States; ^5^Department of Civil and Environmental Engineering, University of New Hampshire, Durham, NH, United States

**Keywords:** natural gas, hydraulic fracturing, dark biosphere, deep subsurface, cultivation, characterization, genome, shale

## Abstract

Hydraulic fracturing is the prevailing method for enhancing recovery of hydrocarbon resources from unconventional shale formations, yet little is understood regarding the microbial impact on biogeochemical cycling in natural-gas wells. Although the metabolisms of certain fermentative bacteria and methanogenic archaea that dominate in later produced fluids have been well studied, few details have been reported on microorganisms prevelant during the early flowback period, when oxygen and other surface-derived oxyanions and nutrients become depleted. Here, we report the isolation, genomic and phenotypic characterization of *Marinobacter* and *Arcobacter* bacterial species from natural-gas wells in the Utica-Point Pleasant and Marcellus Formations coupled to supporting geochemical and metagenomic analyses of produced fluid samples. These unconventional hydrocarbon system-derived *Marinobacter* sp. are capable of utilizing a diversity of organic carbon sources including aliphatic and aromatic hydrocarbons, amino acids, and carboxylic acids. *Marinobacter* and *Arcobacter* can metabolize organic nitrogen sources and have the capacity for denitrification and dissimilatory nitrate reduction to ammonia (DNRA) respectively; with DNRA and ammonification processes partially explaining high concentrations of ammonia measured in produced fluids. *Arcobacter* is capable of chemosynthetic sulfur oxidation, which could fuel metabolic processes for other heterotrophic, fermentative, or sulfate-reducing community members. Our analysis revealed mechanisms for growth of these taxa across a broad range of salinities (up to 15% salt), which explains their enrichment during early natural-gas production. These results demonstrate the prevalence of *Marinobacter* and *Arcobacter* during a key maturation phase of hydraulically fractured natural-gas wells, and highlight the significant role these genera play in biogeochemical cycling for this economically important energy system.

## Introduction

Hydraulic fracturing has enhanced the extraction of oil and gas from previously inaccessible unconventional petroleum resources (e.g., shales, tight sands, coalbeds), with hydraulically fractured wells accounting for the production of 67% of the natural gas and 51% of the oil in the United States in 2015 (Kerr, 2010; Tour et al., 2010; EIA, 2016a,b). Hydraulic fracturing of unconventional systems is accomplished through the high-pressure injection of water (∼90%), proppant (∼9%), and chemical additives (∼1%) into subsurface formations (Arthur et al., 2009; Vidic et al., 2013). This process generates and sustains a network of fractures in the intrinsically low-permeability formation, liberating hydrocarbons which return through the wellbore to be collected at the surface (Arthur et al., 2009; Kerr, 2010; Tour et al., 2010). Injected fluids return to the surface over several months as they reach equilibrium with deep formation brines and undergo water-rock interactions (Vengosh et al., 2017). The fluids that return to the surface are referred to as flowback fluids (generally the first few weeks after hydraulic fracturing occurs) and produced fluids (several weeks to years after hydraulic fracturing occurs and throughout the production life cycle). Flowback fluids are characterized by high carbon concentrations (>100 mg/L), moderate to high salinities (1–8 NaCl%), and increasing concentrations of soluble nitrogen and sulfur species (Chapman et al., 2012; Barbot et al., 2013; Haluszczak et al., 2013; Warner et al., 2013; Cluff et al., 2014; Harkness et al., 2015; Lester et al., 2015). Produced fluids, in contrast, more closely resemble deep formation brines (>10% NaCl). Produced fluids are extremely high in total dissolved solids, have lower carbon content (50 mg/L and below), and contain elevated concentrations of reduced sulfur species (Chapman et al., 2012; Barbot et al., 2013; Haluszczak et al., 2013; Warner et al., 2013; Cluff et al., 2014; Harkness et al., 2015; Lester et al., 2015; Booker et al., 2017; Vengosh et al., 2017). Despite dramatic changes in solution chemistry during the gas extraction process, microbial life has been detected in flowback and produced fluids, and likely impacts fluid chemistry changes, infrastructure stability, and hydrocarbon recovery efficiency (Struchtemeyer and Elshahed, 2012; Cluff et al., 2014; Gaspar et al., 2014; Daly et al., 2016; Liang et al., 2016; Booker et al., 2017; Nixon et al., 2017; Lipus et al., 2018).

Microbial growth in oil and gas wells can contribute to reservoir souring, well corrosion, and clogged pore space, decreasing hydrocarbon productivity (Fichter et al., 2012; Gaspar et al., 2014). Although biocides are commonly added to control microbial growth, evidence of a dynamic bacterial and archaeal community has been observed in fluids collected during and after hydraulic fracturing from multiple unconventional systems in the United States. (Fichter et al., 2012; Struchtemeyer and Elshahed, 2012; Mohan et al., 2013; Cluff et al., 2014; Daly et al., 2016; Lipus et al., 2017, 2018; Borton et al., 2018). These studies suggest that microorganisms are injected from surface input fluids and persist despite extreme environmental conditions such as high salinities, limited access to terminal electron acceptors (e.g., oxygen, nitrate, sulfate), high pressures, and warmer temperatures than near-surface conditions (Mouser et al., 2016). Despite these ecosystem stressors, a low-diversity microbial community persists and plays a significant role in the biogeochemical cycles of unconventional systems, including low porosity shales and limestones (Fichter et al., 2012; Cluff et al., 2014; Daly et al., 2016; Liang et al., 2016; Booker et al., 2017; Nixon et al., 2017; Borton et al., 2018; Lipus et al., 2018).

Prior studies from our group revealed that Marcellus Shale-associated taxa *Halanaerobium, Marinobacter*, and *Frackibacter* may participate in carbon and nitrogen cycling through fermentation/transformation of both injected chemicals and microbially-produced osmoprotectants (Daly et al., 2016). Laboratory studies have suggested that *Halanaerobium* may also contribute to sulfide-induced well-souring and corrosion months to years after fracturing has occurred (Liang et al., 2016; Booker et al., 2017; Nixon et al., 2017; Lipus et al., 2018). The aforementioned studies have focused on taxa that dominate in later production phases characterized by elevated salinity and depleted organic carbon. Still, relatively little has been reported on the physiological and biogeochemical roles of dominant taxa within the first few months of production, a time period that may be critical for the establishment of later persisting taxa. During this natural-gas well maturation transition period, organic carbon is abundant (e.g., indigenous hydrocarbons and injected carbon), salinity is moderate in comparison to later time points (1–10% versus 10–30% in later produced fluids), and terminal electron acceptors (e.g., oxygen, nitrate, sulfate) remain available, allowing for a diversity of microbially-mediated redox reactions and enzymatic transformations not thermodynamically possible during later stages of production (Mouser et al., 2016).

Two genera, *Marinobacter* and *Arcobacter*, have been detected in produced fluids from numerous unconventional hydrocarbon-producing formations including the Barnett, Antrim, Haynesville, Utica, Point Pleasant, and Marcellus (Davis et al., 2012; Fichter et al., 2012; Struchtemeyer and Elshahed, 2012; Mohan et al., 2013; Wuchter et al., 2013; Cluff et al., 2014; Daly et al., 2016; Liang et al., 2016; Mouser et al., 2016). Although one study applied a metagenomics approach to infer *Marinobacter* sp. may be capable of utilizing organic nitrogen and hydrocarbon compounds (Daly et al., 2016), the metabolic capabilities of these taxa in unconventional systems are poorly defined. Here, we used laboratory experiments to investigate salinity tolerance and carbon substrate utilization of *Marinobacter* and *Arcobacter* strains cultivated from natural-gas well produced fluids. We complement these laboratory investigations with genomic, metagenomic, and geochemical data from hydraulically stimulated natural-gas wells, demonstrating the power of using both cultivation and cultivation-independent approaches for elucidating sources and use of carbon and energy for halotolerant microorganisms from a deep terrestrial ecosystem.

## Materials and Methods

### Methods Overview

Fluids were collected from six different hydraulically fractured natural-gas wells for geochemical and genomic analyses summarized in this manuscript: four natural-gas wells producing from the Utica-Pt. Pleasant Formation (Utica-3, Utica-6, Utica-7, and Utica-8), and two natural-gas wells producing from the Marcellus Shale (Marcellus-4 and Marcellus-5) (Table 1). Nitrogen and sulfur analyses were performed on all six wells while metagenomic analyses, near-full-length reconstruction of the 16S rRNA gene, NPOC, and CO_2_ analyses were performed on one well (Marcellus-4). *Marinobacter sp.* UTICA-S1B6 was isolated from Utica-3, whereas *Arcobacter sp.* UTICA-S4D1 was isolated from Utica-8, and *Arcobacter* sp. MARC-MIP3H16 was isolated from Marcellus-4. Additionally, seven 16S rRNA gene sequences reported in a previous study (Marcellus-1, Daly et al., 2016) were used in our phylogenetic analysis.

**Table 1:** Identifiers for hydraulically fractured natural-gas wells analyzed and/or summarized in this study.

Wall Identifier	Formation	Sample days after fracturing	Bacteria isolated	Geochemical analyses performed	Microbial analyses performed	Figures present
Utica-3	Utica-Point Pleasant	38-460	*Marinobacter* sp. UTICA-S1B6	N-NH_3_, TN, S^2−^, ${\text{SO}}_{4}^{2-}$	n/a	Figure 2
Utica-6	Utica-Point Pleasant	38-460	n/a	N-NH_3_, TN, S^2−^, ${\text{SO}}_{4}^{2-}$	n/a	Figure 2
Utica-7	Utica-Point Pleasant	38-460	n/a	N-NH_3_,TN, S^2−^, ${\text{SO}}_{4}^{2-}$	n/a	Figure 2
Utica 8	Utica-Point Pleasant	38-460	*Arcobacter* sp. UTICA-S4D1	N-NH_3_, TN, S^2−^, ${\text{SO}}_{4}^{2-}$	n/a	Figure 2
Marcellus-1	Marcellus	4-328	n/a	n/a	16S EMIRGE	Figure 3
Marcellus-4	Marcellus	24-485	*Arcobacter* sp. MARC-MIP3H16	NH_3_, TN, S^2−^, ${\text{SO}}_{4}^{2-}$, CI^−^, NPOC, CO_2_	Metagenomics, 16S EMIRGE	Figures 2, 3, 5
Marcellus-5	Marcellus	35 496	n/a	NH_3_, TN, S^2−^, ${\text{SO}}_{4}^{2-}$	n/a	Figure 2

### Sample Collection

**FIGURE 1:**
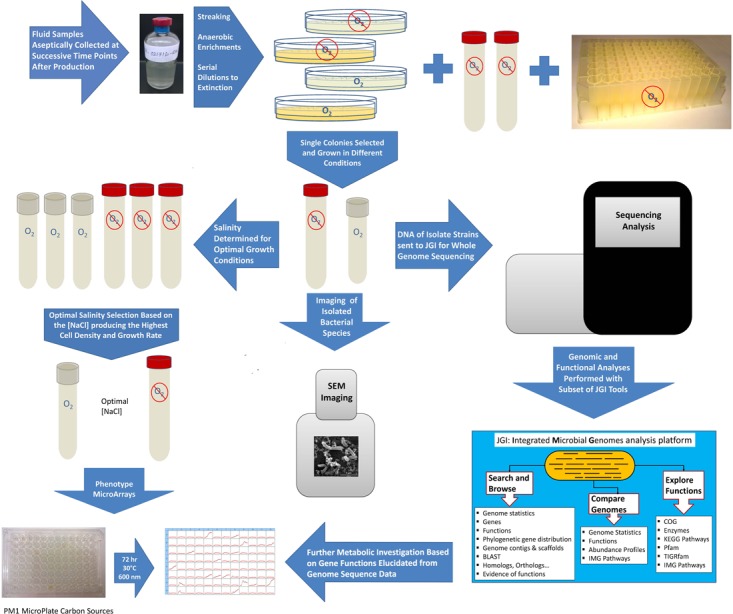
Flowchart summarizing methods used for isolating and characterizing *Marinobacter* and *Arcobacter* strains from produced fluid samples.

Produced fluid samples were collected from six hydraulically fractured natural-gas wells in the northern Appalachian Basin: four from the Utica-Point Pleasant Formation (Utica-3, Utica-6, Utica-7, and Utica-8) and two from the Marcellus Shale (Marcellus-4 and Marcellus-5). Liquid samples were taken from the gas-water separator. Samples were collected from the start of flowback at various intervals over a year into production (496 days) as previously reported (Luek et al., 2018). Samples for bacterial enumeration were collected in 50 mL polypropylene conical vials containing 5.0 mL of 25% paraformaldehyde and stored at 4°C until enumeration. Samples for isolation and culturing (25 mL) were collected in sterile serum bottles with no headspace and sealed with butyl rubber septa to maintain anaerobic conditions, then upon arrival at the laboratory purged with N_2_ gas and stored at ambient temperature in the dark until inoculation (Figure 1). Samples for geochemical analysis were collected in 1 L sterile containers (either HDPE or glass) with no headspace and stored at 4°C until analysis. Sulfide measurements were conducted on unfiltered samples (Hach method 8131, see following section). Samples for total dissolved nitrogen, ammonia, and sulfate were filtered using 0.45 μm PES filters (EMD Millipore, Burlington, MA, United States), with the exception of the Marcellus-4 and Marcellus-5 wells in which 0.22 μm PES filters were used, within 48 h of sampling and stored at 4°C until analysis.

### Non-purgeable Organic Carbon, Nitrogen, Sulfur, and Chloride Analysis

Non-purgeable organic carbon (NPOC) was measured in the Marcellus-4 samples by a TOC/TN analyzer equipped with autosampler (TOC-V CSN/TNM-1/ASI-V, Shimadzu, Kyoto, Japan). Total dissolved nitrogen (TDN) was analyzed in samples from all wells by a Shimadzu TOC/TN analyzer (see above), employing appropriate dilutions as needed for samples. Ammonia (NH_3_) in the produced fluid samples was analyzed colorimetrically using the modified Berthelot reaction method on a Skalar San++ continuous flow nutrient analyzer. Reagents were prepared following the method supplied by the manufacturer. Sulfide was measured using a Hach DR 900, method 8131. Sulfate and chloride were analyzed using a Thermo-Scientific Dionex ICS-2100 ion chromatograph (Waltham, MA, United States); samples were diluted by a factor of 100–1000 due to the high salinity.

### Carbon Dioxide Analysis

Produced gas samples were collected from the Marcellus-4 natural-gas well over a period of 140 days. Produced gas samples were collected with negligible air contamination using thick-walled 0.95 cm (3/8 inch) outside diameter and 40.6 cm (16 inch) long refrigeration-grade copper tubes. The samples were collected by pumping produced fluid that had been collected using HDPE carboys from the producing natural-gas well through the copper tubes, with tubes tapped to remove air bubbles prior to collection. The copper tubes were then sealed using brass refrigeration clamps with a 0.762 mm (0.030 inch) gap (Kang et al., 2016; Harkness et al., 2017). Gas samples in the copper tubes were prepared for analysis by cold welding ∼2.5 cm (∼1 inch) splits of the copper tubing using stainless steel clamps. The copper tube was then attached to an ultra-high vacuum steel line (total pressure = 1–3 × 10^−9^ torr), which is monitored continuously using a 0–20 torr MKS capacitance monometer (accurate to the nearest thousandths) and an isolated ion gauge, using a 0.64 cm (1/4 inch) VCR connection and expanded to obtain aliquots for various gas geochemical analyses.

Carbon dioxide concentrations (CO_2_) were measured on a SRS Quadrupole MS and an SRI 8610C Multi-Gas 3+ gas chromatograph (GC) equipped with a flame ionization detector (FID) and thermal conductivity detector (TCD) at the Ohio State University Noble Gas Laboratory (Darrah et al., 2015; Heilweil et al., 2016; Moore et al., 2018). All reported CO_2_ concentrations were above the method detection limit. The average external precision was determined by measuring a series of synthetic natural gas standards obtained from Praxair and the DCG Partnership. Standard analytical errors for CO_2_ was less than ±1.06% based on daily replicate measurements during analyses.

### Microbial Enumeration

Microbial (bacterial and archaeal) enumeration was performed using filtration and epifluorescent microscopy as described previously (Noble and Fuhrman, 1998; Weinbauer et al., 1998; Chen et al., 2001; Brussaard, 2004; Patel et al., 2007; Diemer et al., 2013). For each sample, the volume filtered was optimized such that at least two filters were prepared to yield approximately 10–200 cells per counting field. Between 1 and 15 mL of fixed sample was filtered through a 25 mm dia. 0.2 μm pore black PCTE filter (Sterlitech, Kent, WA, United States), stained with 2X SYBR Gold (Life Technologies, Carlsbad, CA, United States) in TE buffer, then mounted on a microscope slide with SlowFade reagent (Life Technologies, Carlsbad, CA, United States). The slide was viewed with a Labomed Lx500 epifluorescent microscope through a 40X air objective under 480 nm excitation. For each sample 20 randomly selected fields were counted per filter.

### Bacterial Isolation

*Marinobacter* sp. UTICA-S1B6 was recovered from produced fluid collected from a Utica-Point Pleasant natural-gas well (Utica-3) on the first day of flowback using Difco Marine Broth 2216 (DM2216) medium supplemented with 40 mM nitrate at 30°C (Figure 1, Tummings et al., 2018). *Arcobacter* strains UTICA-S4D1 and MARC-MIP3H16 were isolated from produced fluids collected at 159 days (from the Utica-Point Pleasant formation, Utica-8) and 93 days (from Marcellus Shale, Marcellus-4) after hydraulic fracturing began, respectively (Figure 1, Panescu et al., 2018). *Arcobacter* strains were grown at 30°C in the dark on DM2216 at 5% NaCl purged with 80:20 N_2_/CO_2_. We tested growth under anaerobic and aerobic conditions; because cells grew better in the presence of oxygen, they were maintained under aerobic conditions for growth experiments (Figure 1). The purity of isolates was verified by 16S rRNA sequencing (see *Isolation and Sequencing of DNA*).

### Salinity Growth Curves

Two of the three isolates, *Marinobacter sp*. UTICA-S1B6 and *Arcobacter sp.* MARC-MIP3H16, were tested for salinity tolerance (Figure 1 and Supplemental Table S2). Salinity experiments for both strains were conducted aerobically at 30°C in the dark with shaking. In addition, an anaerobic experiment was also carried out for *Marinobacter* at 37°C in the dark. For *Arcobacter*, a stock of Trypticase Soy Broth (Cat. No. 1.05459, EMD Millipore Corporation, Billerica, MA, United States) was amended with 1% DL Trace Mineral Solution and NaCl at the following concentrations: 0.5, 2, 4, 6, 8, 10, 12, and 14%. For *Marinobacter*, bacterial growth medium consisting of 25% DM2216 and 75% Lennox Broth (Bonis and Gralnick, 2015) with 1% NaCl, was prepared from commercially available powdered mixes. To these media, NaCl was added to obtain the following final concentrations: 0.9, 2.5, 5, 7.5, 10, 12.5, 15, and 20%. Singlet cultures were grown to mid-log phase, 2% was transferred into fresh media, then 2% was transferred again into triplicate cultures from which data were used for the salinity curves. The media was aliquoted into 9 mL increments in borosilicate glass tubes suitable for anaerobic culturing of size 18 × 150 mm purchased from Bellco Glass (Vineland, NJ, United States) and autoclaved at 121°C for 20 min; the tubes were used for both the aerobic and anaerobic cultures. Aerobic media tubes were covered with silicone foam stoppers while the anaerobic media tubes were closed with butyl rubber stoppers clamped with aluminum seals. Absorbance at a wavelength of 610 nm was measured with a Hach DR 900 Colorimeter (Loveland, CO, United States) fitted with a custom adapter.

A standard curve relating optical density to cell numbers was constructed. Cultures were grown to late log phase in the same test media at 2.5% NaCl, fixed (2.5% paraformaldehyde final concentration) and diluted or concentrated to obtain absorbance values of <0.1, ∼0.5, and ∼1.0 at 610 nm. Two aliquots from each concentration were diluted in 2.5% NaCl to yield 10–200 cells per counting field, and counted exactly as described above in the Section “*Microbial Enumeration*.” Growth rates were calculated using a first-order model based on cell numbers and time elapsed.

### Utilization of Carbon Substrates

Culture plates containing DM2216 with 2% agar (Affymetrix USB, Tewksbury, MA, United States) were streaked with approximately 10 μL of cryogenic glycerol stock containing bacterial isolates and incubated at 30°C for 2–3 days. Individual 1–2 mm diameter colonies were picked, resuspended in liquid DM2216, re-streaked and re-grown as described above. Second generation colonies were resuspended and inoculated in Biolog PM1 (Hayward, CA, United States) inoculating fluid augmented with 5% NaCl solution, 20 μM pyruvate (carbon source) and 0.12X Marinobacter Iron Medium (MIM) (Bonis and Gralnick, 2015, AffymetrixUSB, Tewksbury, MA, United States) (Figure 1). The cultures were grown to late log phase (2–3 days) where 2% was transferred into fresh PM1-MIM-pyruvate medium. Once the second generation PM1-MIM-pyruvate culture reached the desired density, the cells were pelleted for 10 min at 5,000 × *g*, washed twice in 5% NaCl, and resuspended in 500 μL PM1-MIM with no carbon source. The washed culture was quantified on a NanoDrop 2000 spectrophotometer (Thermo Scientific) at 600 nm in an Eppendorf Uvette fitted with an adapter (50 μL of culture used), and diluted to 0.1 Absorbance units with PM1-MIM, of which 880 μL was combined with an additional 12 mL PM1-MIM inoculating fluid and distributed to a new PM1 substrate plate at 110 μL per well. All carbon substrate assessments were performed under aerobic conditions. The plate was sealed with a sterile, optically clear, non-cytotoxic, gas-permeable adhesive film (4titude, Wotton, England). Optical densities were measured using a plate reader (BioTek Synergy HTX Multi-Mode Reader, Winooski, VT, United States) at 600 nm every 2 h for 3 days while incubating at 30°C. Normalization took place by subtracting the optical densities of a control plate containing media with no cells.

### Isolation and Sequencing of DNA

*Marinobacter* and *Arcobacter* genomic DNA was extracted using Qiagen DNA extraction kits (Qiagen DNA minikit and Qiagen DNeasy kit, respectively) (Hilden, Germany) as previously described (Panescu et al., 2018; Tummings et al., 2018). The 16S rRNA gene was amplified with universal primer sets for bacteria (27F, 1492R) and archaea (4Fa, 1492R). The 16S rRNA gene was sequenced using the Sanger method on a 3730 DNA Analyzer (Applied Biosystems) for quality control at the Comprehensive Cancer Center Genomics Shared Resource at the Ohio State University. After passing quality control, the isolates were subjected to full genome sequencing using an Illumina MiSeq at the Joint Genome Institute (JGI), Walnut Creek, CA, United States. Genome assemblies were constructed using Spades v. 3.6.2 with annotation performed by the JGI in the Integrated Microbial Genomes platform v. 4.12.1 (Figure 1, Huntemann et al., 2016; Panescu et al., 2018; Tummings et al., 2018).

### Phylogenetic Analysis

Maximum-likelihood (ML) phylogenetic trees were constructed in RAxML using the full length 16S rRNA gene in Geneious v. 8 using a MUSCLE alignment with a maximum of 10,000 iterations (v.7.2.8, nucleotide model GTR gamma, rapid bootstrapping and search for best-scoring ML tree algorithm, with 1,000 bootstrap replicates). Nearly full-length 16S rRNA EMIRGE sequences used in the trees were reconstructed from related shale fluid metagenome samples as described previously (Miller et al., 2011; Daly et al., 2016).

### Scanning Electron Microscopy (SEM) Sample Preparation and Imaging

Cells from cultures grown to log phase were fixed by combining 178 μL culture with 22 μL 25% PFA and allowed to equilibrate for 1–2 h in a microcentrifuge tube. Cells were next pelleted at 5,000 × *g* for 10 min, supernatant was removed, and cell pellet was washed with 1.0 mL PBS and resuspended in 200 μL PBS. For each isolate, ∼100 μL of the resuspended culture was placed on a 0.2 μm Nucleopore polycarbonate filter (Whatman, GE Healthcare, Chicago, IL, United States) mounted on a 25 mm diameter Swinnex syringe filter holder (EMD Millipore, Darmstadt, Germany) connected to flexible tubing. The culture was embedded into the filter by applying gentle vacuum (manually with a disposable syringe or by using an electric vacuum pump). The filter was next rinsed with 300–500 μL of PBS at 2% NaCl followed by additional vacuum application. After the culture was embedded onto the filter and all excess liquid was removed, the filter was allowed to dry. Dried samples were subjected to successive 5 min rinses of increasing concentrations of 35–100% ethanol. After evaporation, the filter was covered in hexamethyldisilazane, covered with a petri dish lid and allowed to dry overnight. The filters were subsequently mounted with carbon tape on aluminum stubs and imaged with a FEI Quanta FEG 250 scanning electron microscope at the Subsurface Energy Materials Characterization and Analysis Laboratory (SEMCAL), School of Earth Sciences, The Ohio State University (Figure 1).

### Metagenomic Sequencing, Assembly, and Annotation

Metagenomic sequencing, assembly, and annotation was performed as previously described (Borton et al., 2018). In short, produced fluid samples collected at 7 time points (drilling mud sample prior to fracturing, days after fracturing 25, 34, 35, 79, 93, 142) from Marcellus Shale natural-gas well Marcellus-4 (300–1000 ml) were concentrated on a 0.22-μm PES filter (Nalgene; Fisher Scientific). Total nucleic acids were extracted using a modified phenol-chloroform extraction method. Briefly, libraries were created and quantified using an Illumina Library creation kit (KAPA Biosystems) with solid-phase reversible 402 immobilization size selection. Libraries were then sequenced on the Illumina HiSeq 2500 sequencing platform utilizing a TruSeq Rapid paired-end 404 cluster kit. Fastq files were generated with CASSAVA 1.8.2. Illumina sequences from each sample were first trimmed from both the 5′ and 3′ ends with Sickle and then each sample was assembled individually with IDBA-UD (Wrighton et al., 2012; Brown et al., 2015) using default parameters. Scaffolds were annotated as described previously by predicting open reading frames with MetaProdigal (Hyatt et al., 2012). Sequences were compared with USEARCH (Edgar, 2010) to KEGG, UniRef90, and InterProScan (Quevillon et al., 2005) with single and reverse best hit (RBH) matches of >60 bases reported.

### Genomic and Metagenomic Analyses

Inquiry into genes contained in isolate genomes was performed using IMG/MER from JGI (Figure 1, Chen et al., 2017). Genes in isolate genomes were pulled from IMG/MER using EC numbers and KEGG Orthology numbers after genomic annotation following the JGI genome annotation pipeline (Huntemann et al., 2016). Homologous genes in assembled metagenomes were mined by KEGG orthology number, EC number, and name with a cutoff of >200 bit score and >45.9% identity similarity to genus *Marinobacter/Arcobacter* at day 34 (after fracturing) for *Marinobacter* and *Arcobacter* and days 34 and 93 for *Arcobacter* (Booker et al., 2017). *Arcobacter* and *Marinobacter* normalized relative abundances through time (days 25, 34, 35, 79, 93, 142) were tracked using the 16S rRNA gene reconstructed from the metagenomic data using EMIRGE (Miller et al., 2011; Daly et al., 2016).

### Data Accession

Whole genome sequences for *Arcobacter* sp. MARC-MIP3H16 and UTICA-S4D1 were deposited in NCBI under accession numbers PTIW01000000 and FUYO01000000 and at the JGI IMG/M database under genome IDs 2700989666 and 2703719342, respectively (Pansecu et al., 2018). Whole genome sequences for *Marinobacter* sp. UTICA-S1B3, UTICA-S1B6, UTICA-S1B9 are available in NCBI under accession numbers PTIV00000000, PTIT00000000, and PTIU00000000, respectively, and at JGI IMG/M database under genome IDs 2700989663, 2700989662, and 2700989665 (Tummings et al., 2018). All metagenomic sequencing data has been deposited under Bioproject PRJNA308326.

## Results

### *Marinobacter* and *Arcobacter* Persist in Unconventional Source Ecosystem for Weeks to Months After Hydraulic Fracturing

**FIGURE 2:**
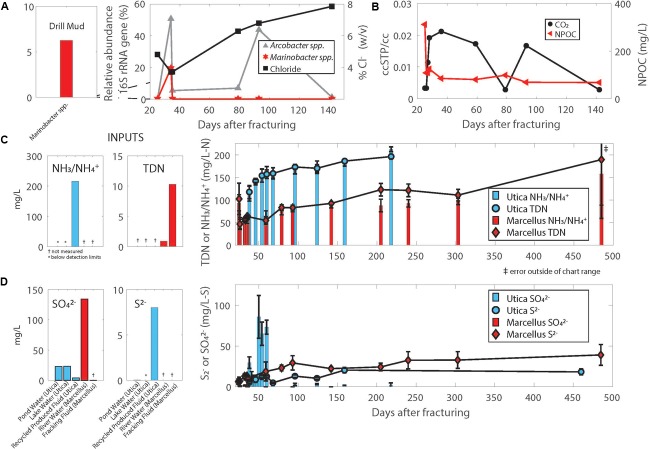
**(A)** Relative abundance of the 16S rRNA gene corresponding to *Arcobacter* and *Marinobacter* reconstructed from metagenomes using EMIRGE combined with chloride trends in the same Marcellus Shale natural-gas well (Marcellus-4). **(B)** CO_2_ and NPOC (non-purgeable organic carbon) trends in the Marcellus Shale natural-gas well (Marcellus-4). Temporal trends in nitrogen **(C)** and sulfur **(D)** in produced fluids from Utica-Point Pleasant formation and Marcellus Shale natural-gas wells. Plotted values are averages of 4 Utica-Point Pleasant natural-gas wells (Utica-3, Utica-6, Utica-7, Utica-8) and 2 Marcellus natural-gas wells (Marcellus-4 and Marcellus-5); error bars are standard deviations between measurements for Utica wells, range for Marcellus wells. Averages were calculated using the same day after flowback began and converted to days after fracturing. Offset graphs to the left of main graphs indicate measurements in drill muds, injected fluid, or source waters. NH_3_/${\text{NH}}_{4}^{+}$ indicates total ammonia/ammonium which are both measured in this method.

**FIGURE 3:**
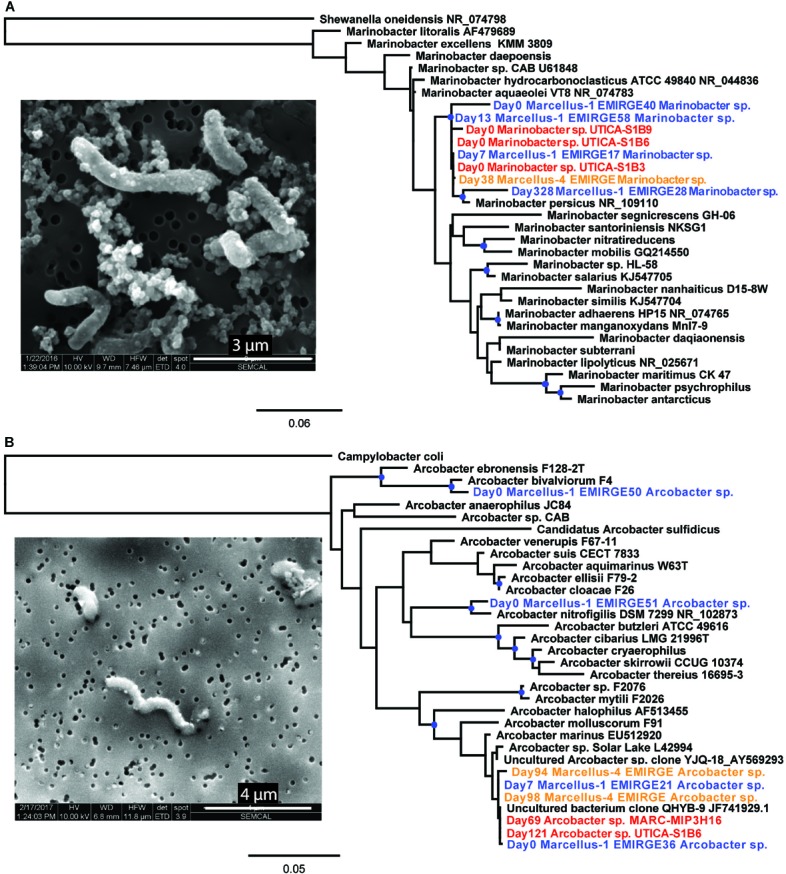
SEM images of **(A)** *Marinobacter* sp. UTICA-S1B6 and **(B)** *Arcobacter* sp. MARC-MIP3H16 and phylogenetic placement of **(A)** *Marinobacter* sp. UTICA-S1B6 and **(B)** *Arcobacter* spp. MARC-MIP3H16 and UTICA-S4D1. Orange text denotes near-full-length 16S rRNA gene sequences reconstructed from Marcellus Shale natural-gas well Marcellus-4 metagenomes using EMIRGE; blue text denotes near-full-length 16S rRNA gene sequences reconstructed from metagenomes using EMIRGE in a previous study, Marcellus-1; red text denotes full-length 16S rRNA gene sequences from Marcellus or Utica Pt. Pleasant natural-gas well isolates. Branches marked with blue dots indicate bootstrap support greater than or equal to 80%.

Using near-full-length 16S rRNA gene sequences reconstructed from 7 sample metagenomes using EMIRGE, we tracked the relative abundance of two key taxa, *Marinobacter* and *Arcobacter*, during the first 140 days after hydraulic fracturing in a Marcellus Shale natural-gas well (Marcellus-4). *Marinobacter* were detected in drill muds (6.3%) used during natural-gas well development, and produced fluid samples collected in the first 4 weeks after hydraulic fracturing, reaching up to 20% relative abundance on day 34, but decreased thereafter to levels below 0.5% (Figure 2A). We compared a representative 16S rRNA gene sequence reconstructed from metagenomes in the Marcellus natural-gas well (Marcellus-4) using EMIRGE (Day 38 EMIRGE *Marinobacter* sp, Figure 3A) to (i) near-full-length 16S rRNA gene sequences reconstructed from metagenomes in another previously studied Marcellus Shale natural-gas well (Marcellus-1) (Figure 3A, Daly et al., 2016), (ii) 16S rRNA gene sequences from genomes available for *Marinobacter* strains isolated from the Utica-Point Pleasant Formation (Utica-3) (Day 0 after flowback began), and (iii) 16S rRNA gene sequences acquired from NCBI for *Marinobacter* strains isolated from other marine environments. Representative *Marinobacter* sampled from Marcellus-4 were highly similar to the three isolates recovered from the Utica-Pt. Pleasant Formation (97–99% shared nucleotide identity) (UTICA-S1B3; UTICA-S1B6; UTICA-S1B9) and cluster closely to 16S rRNA gene sequences collected from another Marcellus Shale natural-gas well (Marcellus-1, Figure 3A).

Based on visual analysis of SEM micrographs, *Marinobacter* sp. UTICA-S1B6 is rod-shaped and approximately 2–4 μm long by 0.5 μm thick (Figure 3A). The closest related strain to *M.* UTICA-S1B6*, Marinobacter persicus*, is a strict aerobe isolated from a hypersaline lake and capable of tolerating salinities up to 20% NaCl (Bagheri et al., 2013). Other closely related strains *M. aquaeolei* and *M. hydrocarbonoclasticus* were discovered in environments with high concentrations of hydrocarbons (oil well, marine petroleum spill, respectively) (Gauthier et al., 1992; Huu et al., 1999).

*Arcobacter* spp. generally persist in the first weeks to months after hydraulic fracturing and remain abundant for a longer time period than *Marinobacter* before replacement by other abundant taxa commonly identified in produced fluids including *Halanaerobium* and *Methanohalophilus* as natural gas and produced fluid production continues and the natural-gas well matures (Davis et al., 2012; Mohan et al., 2013; Wuchter et al., 2013; Cluff et al., 2014; Liang et al., 2016). Although we did not detect *Arcobacter* in drill muds, it comprised over 50% of the microbial community in produced fluids from the Marcellus-4 natural-gas well collected on day 34, and subsequently ranged in relative abundance from 1 to 44% until day 142 (Figure 2A). In a similar fashion to *Marinobacter* discussed above, we compared these *Arcobacter* 16S rRNA sequences reconstructed from the Marcellus-4 natural-gas well metagenomes using EMIRGE (Days 94, 98 EMIRGE *Arcobacter* sp.) to (i) near full-length 16S rRNA sequences reconstructed from metagenomes in another previously studied Marcellus Shale natural-gas well (Marcellus-1) (Days 0, 7, Figure 3B, Daly et al., 2016), (ii) 16S rRNA gene sequences for two *Arcobacter* strains isolated from the Utica-Point Pleasant Formation (Utica-8) (Day 159 after hydraulic fracturing occurred) and Marcellus Shale natural-gas well Marcellus-4 (Day 93 after hydraulic fracturing), and (iii) 16S rRNA gene sequences acquired from NCBI for *Arcobacter* strains isolated from other marine environments. Representative *Arcobacter* sequences recovered from both Marcellus-4 (Day 94 Marcellus-4 and Day 98 Marcellus-4) and Marcellus-1 (T0_EMIRGE36 and T7_EMIRGE21) were very similar to *Arcobacter* spp. MARC-MIP3H16 and UTICA-S4D1 isolates. In particular, sequence from an initial flowback sample (Day 0 Marcellus-1) and an uncultured clone were most closely related (99.6%) to laboratory cultured strains (Figure 3B).

*Arcobacter* sp. MARC-MIP3H16 is a curved rod approximately 1–3 μm long by 0.3–0.5 μm thick with a polar flagellum (not shown) (Figure 3B). The closest related strains to *A.* MARC-MIP3H16, *Arcobacter* sp. *solar lake* and *Arcobacter marinus*, were both isolated from saline aquatic environments (Donachie et al., 2005; Kim et al., 2010). *Arcobacter marinus* can grow in 3–5% NaCl under both aerobic and microaerophilic conditions. Another closely related strain, *Arcobacter halophilus*, isolated from a hypersaline lagoon, is capable of growth across aerobic to anaerobic conditions, provided media contains at least 2% NaCl (Donachie et al., 2005). The close clustering of isolate and sample DNA despite lithogical and geographical differences demonstrates the prevalence of both *Marinobacter* and *Arcobacter* in early produced fluid from hydraulically fractured natural-gas wells.

### Shale *Marinobacter* and *Arcobacter* Are Slight to Moderate Halophiles

**FIGURE 4:**
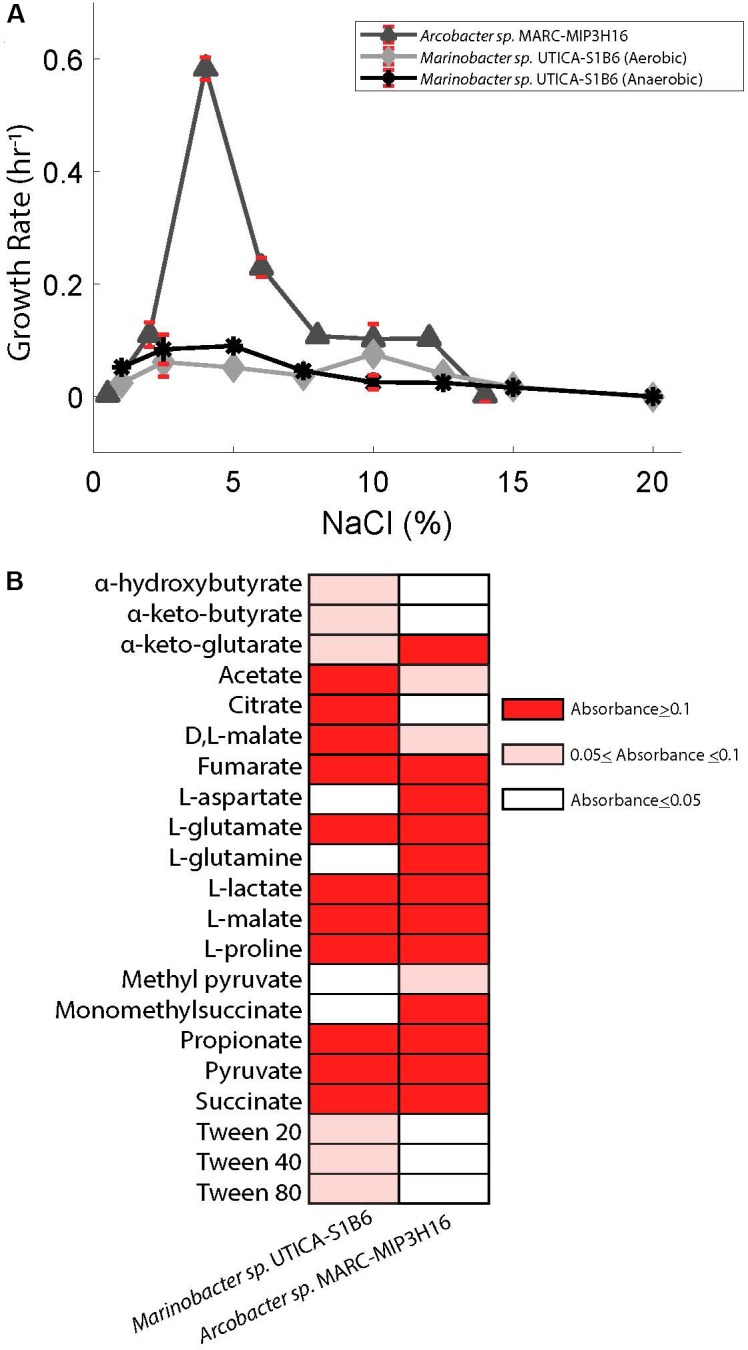
**(A)** Superimposed salinity growth rate curves for *Arcobacter* sp. MARC-MIP3H16 and *Marinobacter* sp. UTICA-S1B6. Error bars denote standard deviation from triplicate measurements. **(B)** Carbon sources utilized by *Marinobacter* sp. UTICA-S1B6 and *Arcobacter* sp. MARC-MIP3H16. Negative controls registered absorbance values of 0.023 and 0.014, respectively. Any absorbance readings under 0.05 were considered negative and assigned a white color in this figure.

The highest relative abundance of 16S rRNA gene copy numbers detected for *Marinobacter* and *Arcobacter* was associated with lower salt concentrations in the Marcellus-4 natural-gas well (approximately 40 g/L Cl^−^ or 4% Cl^−^, Figure 2A). Therefore, to better understand the adaptability of *Marinobacter* and *Arcobacter* to increasing salinity levels, we tested the tolerance of both isolates across a broad range of salinities under aerobic (*Arcobacter, Marinobacter)* and anaerobic (*Marinobacter)* conditions (Figure 4A). *Marinobacter* sp. UTICA-S1B6 grew in concentrations between 0.9 and 15% Cl^−^, with optimal growth at 10% Cl^−^ when oxygen was present (Figure 4A). In the absence of oxygen, *Marinobacter* growth rate improved at a lower salinity (5% Cl^−^). *Arcobacter* sp. MARC-MIP3H16 grew in salt concentrations between 2 and 12%, with an optimal growth rate at 4% Cl^−^ (0.6 hr^−1^), considerably higher than growth at all other salinities (<0.25 hr^−1^) (Figure 4A). Our laboratory findings were therefore consistent with field salinity levels when *Marinobacter* and *Arcobacter* were highest in relative abundance during the first 140 days of production in the Marcellus-4 natural-gas well (4–8%). However, *Arcobacter* growth rate was significantly higher than *Marinobacter* in this range, which may partially explain its dominance and persistence during this transitional period in Marcellus-4 natural gas and produced fluid well maturation.

**FIGURE 5:**
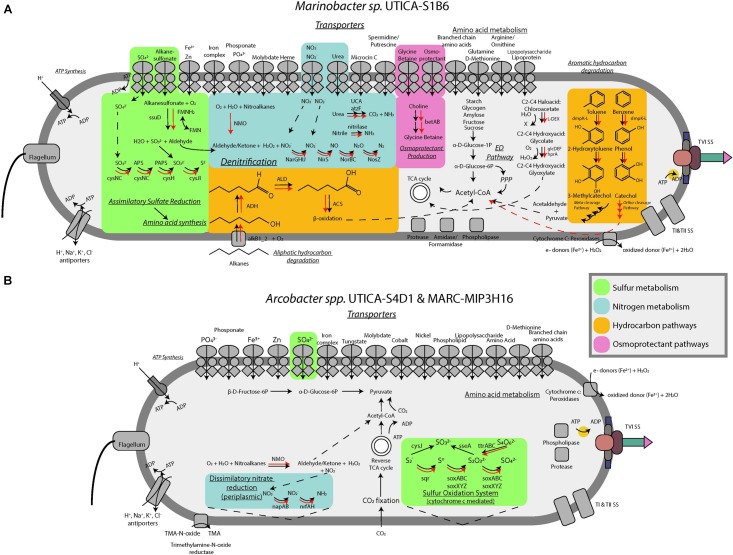
Conceptual metabolic models for **(A)** *Marinobacter* and **(B)** *Arcobacter* isolates incorporating isolate genomic (black arrows) and metagenome data (red arrows).

To assess possible osmoadaptation mechanisms, we searched the isolate genomes for relevant genes associated with osmoprotectant synthesis and transport, and the movement of ions across the cell membrane (Daly et al., 2016). Genomic evidence of the isolated culture indicated that *Marinobacter.* sp. UTICA-S1B6 has a glycine betaine transporter for osmolyte uptake as well as the dehydrogenases responsible for conversion of choline into glycine betaine under aerobic conditions for *de novo* osmoprotectant synthesis. We also discovered several genes responsible for the synthesis of ectoine, L-ectoine synthase (*ectC*) and ectoine hydroxlase (*ectD*), and anti-porter genes (H^+^, Na^+^, K^+^, Cl^−^) responsible for the osmotic regulation within the cell (Figure 5, Oren, 2008). *Arcobacter spp.* UTICA-S4D1 and MARC-MIP3H16 both have the putative ability to synthesize ectoine via *ectD*, but not *ectC*. These *Arcobacter* strains do not have the functional potential for synthesis of osmolytes other than ectoine, but instead contain anti-porters (H^+^, Na^+^, K^+^, Cl^−^) that may be used for adaptation to changing concentrations of NaCl via the “salt-in” strategy, as well as transporters for glycine betaine and polar amino acids for uptake of extracellular osmolytes (Figure 5, Oren, 2008; Daly et al., 2016). Neither *Marinobacter* sp. UTICA-S1B6 nor *Arcobacter spp.* UTICA-S4D1 and MARC-MIP3H16 have genes responsible for uptake or synthesis of osmolytes sorbitol/mannitol, or trehalose.

### *Marinobacter* and *Arcobacter* Utilize a Wide Variety of Carbon Sources

*Marinobacter* sp. UTICA-S1B6 can utilize a variety of organic compounds for carbon and energy sources, as evidenced by isolate genomic potential and laboratory physiological assessments. We detected genes for the Entner-Doudoroff pathway for conversion of glucose to pyruvate in *M*. UTICA-S1B6, however, this pathway was not complete as it lacked all genes responsible for phosphorylation of glucose (EC 3.1.3.9, 2.7.1.1, 2.7.2.2, and 2.7.1.199). Consistent with our genomic inferences, we confirmed the isolate’s inability to grow on glucose under oxic laboratory conditions (Supplementary Figure S1). We detected multiple amino acid ABC transporters and di/tricarboxylate transporters in the *Marinobacter* isolate genome, providing evidence that amino acid uptake is important for this strain. In complementary laboratory experiments, *Marinobacter* *sp.* UTICA-S1B6 grew on two amino acids (L-glutamate, L-proline) and several carboxylic acids (OD>0.1) as the sole carbon source, with citrate, L-malate, and acetate as three of the more optimally utilized compounds (Figure 4B). In addition, genes responsible for breakdown and utilization of starch, glycogen, amylose, fructose, and sucrose are present in the isolate genome (Figure 5A); however, *M.* UTICA-S1B6 did not grow on sucrose or fructose as a sole carbon source in the laboratory (Supplementary Figure S1).

Our genomic investigation also showed that *Marinobacter* sp. UTICA-S1B6 has the potential to degrade aliphatic and aromatic hydrocarbons. The strain contains the alkane-1-monooxygenase gene (*alkB)* responsible for activating alkanes to a primary alcohol using oxygen, as well as the alcohol and aldehyde dehydrogenases that would be necessary for substrate transformation before β-oxidation and processing through the TCA cycle (Figure 5A). Although we did not test the strain’s growth on aliphatic hydrocarbons, *M.* UTICA-S1B6 grew at mid-range optical densities (0.05–0.1) on polysorbate non-ionic surfactants Tween 20, 40, and 80, which contain long-chain hydrocarbons (Figure 4B). Genes known to catalyze the anaerobic breakdown of hydrocarbons (benzylsuccinate synthase, alkylsuccinate synthase) through fumarate addition (Agrawal and Gieg, 2013) were not identified in the genome, making it unlikely that *Marinobacter* sp. UTICA-S1B6 degrades hydrocarbons in the absence of oxygen.

Besides aliphatic hydrocarbons, *Marinobacter* sp. UTICA-S1B6 has the putative ability to degrade benzene and toluene, both of which have been found in produced fluids from the Marcellus Shale at concentrations in the low part per million range (Hayes et al., 2012). The pathway involving benzene and toluene degradation employs the *dmpK-L* gene family, found in the *M.* UTICA-S1B6 genome, which converts benzene to phenol and later to catechol, or toluene to 2-hydroxytoluene and later 3-methylcatechol (Kanehisa and Goto, 2000). Catechol and 3-methylcatechol are further metabolized by the *xylE-J* gene family to acetaldehyde and pyruvate, both of which can be converted to acetyl-CoA and into central metabolism (e.g., the TCA cycle) (Kanehisa and Goto, 2000). This strain does not have the capacity to utilize other common aromatic compounds such as xylene, ethylbenzene, or PAHs (e.g., naphthalene). Additionally, we found a haloacid dehalogenase gene (L-DEX) in the *M.* UTICA-S1B6 genome, encoding for the conversion of (S)- 2-haloacids (e.g., haloacetates, halopropionates, halobutyrates containing fluoro, chloro, bromo, or iodo substiutions) to (R)-2-hydroxyacids (Goldman et al., 1968). Production of 2-hydroxyacids such as glycolate could be further broken down into glyoxylate, which can be utilized in the TCA cycle (Figure 5A). The genes responsible for this pathway, *glcDEF* and *hprA*, were detected in the *M.* UTICA-S1B6 genome as well as the Marcellus-4 metagenomes (Figure 5A).

Our genomic investigation revealed *Arcobacter* *spp.* MARC-MIP3H16 and UTICA-S4D1 may fix CO_2_ via the reverse TCA cycle, in a similar fashion to other reported *Arcobacter* strains (Figure 5A) (Wirsen et al., 2002; Hügler et al., 2005; Klatt and Polerecky, 2015; Roalkvam et al., 2015). These two *Arcobacter* strains contained all nine enzymes involved in the reductive TCA cycle (Hügler et al., 2005). We followed trends in both organic [non-purgeable organic carbon (NPOC)] and inorganic carbon in the form of CO_2_ gas from the Marcellus-4 natural-gas well. Both CO_2_ and NPOC fluctuate over time, but there is a strong association between higher CO_2_ concentrations and higher relative abundance of *Arcobacter* based on the 16S rRNA gene around day 35 and 80 (Figures 2A,B). Although growth on inorganic carbon was not tested with these *Arcobacter* isolates, and CO_2_ trends in natural-gas wells are likely influenced by a variety of biogeochemical factors, this association suggests higher concentrations of CO_2_ may enable growth of *Arcobacter* through an autotrophic metabolism or chemosynthesis of reduced sulfur species during certain times of production.

In addition to the genomic potential to fix inorganic carbon, *Arcobacter* sp. MARC-MIP3H16 utilized 25 different amino acids and carboxylic acids (Figure 4B). In the genome, we found amino acid ABC transporters, monosaccharide transporters, dicarboxylate transporters, and C4-transporters, confirming the ability to uptake these compounds. Amino acids L-glutamine, L-glutamate, L-aspartate, and L-proline were optimally utilized as sole carbon sources, (OD>0.1). The carboxylic acids L-malate, propionate, pyruvate, L-lactate, and succinate were optimally converted for growth as sole carbon sources while several other carboxylic acids were minimally utilized (optical density changes between 0.05 and 0.1), such as acetate and methyl pyruvate.

### *Marinobacter* and *Arcobacter* Influence Nitrogen, Sulfur, and Iron Cycles in Unconventional Systems

We tracked total dissolved nitrogen (TDN) and ammonium/ammonia in six northern Appalachian Basin natural-gas wells and observed a consistent trend after hydraulic fracturing. TDN levels increased from under 5 mg/L in initial flowback samples to above 100 mg/L within 6 months of production in natural-gas wells from both the Utica-Point Pleasant Formation and Marcellus Shale (Figure 2C). The majority of TDN (59% or greater at all time points) was comprised of fixed sources of nitrogen (ammonia/ammonium). Nitrate was analyzed but was below detection (<1 ppm N) at all time points in flowback and produced fluid samples. Other possible sources of nitrogen in the system include organic nitrogen compounds, which were not measured.

Although nitrate was not detected in ppm concentrations for these natural-gas wells, nitrate reduction is a possible mode of energy generation for both *Marinobacter* and *Arcobacter*, especially at levels below our analytical detection limits. Our genomic analysis showed that *Marinobacter* sp. UTICA-S1B6 is capable of denitrification, possessing the proper transporters and catalytic genes for complete conversion of nitrate to N_2_ (Figure 5A). In addition to utilizing inorganic nitrogen as an electron acceptor, *M.* UTICA-S1B6 can convert urea to ammonia with urease, which is especially interesting given urea is a component in the hydraulic fracturing fluid formulation for one of the Marcellus natural-gas wells in this study (Supplementary Figure S2). *Arcobacter* spp. MARC-MIP3H16 and UTICA-S4D1, on the other hand, are capable of dissimilatory nitrate reduction to ammonia. Like *Marinobacter* sp. UTICA-S1B6, *Arcobacter* spp. MARC-MIP3H16 and UTICA-S4D1 can also oxidize nitroalkanes via the *NMO* gene, which may serve as an alternative source of nitrate within the cell.

The genome of *Marinobacter* sp. UTICA-S1B6 contains genes responsible for importing and assimilating sulfur from both inorganic (e.g., sulfate) and organic sulfur forms. Specifically, *M.* UTICA-S1B6 can putatively utilize alkanesulfonates via the *ssuD* gene, producing sulfite and an aldehyde group (Figure 5A) (Eichhorn et al., 1999). The ability to convert alkane-derived nutrients to sources of carbon and macronutrients (e.g., sulfate, nitrate) is consistent in the Marcellus-4 metagenomes that cluster closely to our *Marinobacter* isolate (Figure 5A and Supplementary Table S1).

One of the more unique aspects of *Arcobacter* *sp.* MARC-MIP3H16 relative to other taxa derived from these hydrocarbon bearing systems is its ability to completely oxidize reduced sulfur compounds (*inc.* sulfide, elemental sulfur, and thiosulfate) to sulfate using the *sox* pathway (Figure 5B). Like other *Arcobacter*, *A.* MARC-MIP3H16 may be coupling this electron transfer process to chemosynthesis (Friedrich et al., 2005; Klatt and Polerecky, 2015; Roalkvam et al., 2015). Our geochemical analysis of sulfate and sulfide in produced fluids from natural-gas wells in both the Utica-Point Pleasant Formation and Marcellus Shale supports the potential for these metabolisms to occur in these systems. Sulfate was present at part per million concentrations (20–140 mg/L) in source waters used for hydraulic fracturing of these natural-gas wells (Figure 2D). In the Marcellus natural-gas wells, within 10 days after hydraulic fracturing, sulfate dropped below 1 mg/L and remained below detection thereafter (Figure 2D). In the Utica-Point Pleasant natural-gas wells, sulfate concentrations varied between 11 and 60 mg/L during the first 59 days post-fracturing. After this time, sulfate concentrations decreased to below 6 mg/L while sulfide increased to between 10–22 mg/L until about day 150 after hydraulic fracturing. Sulfide levels plateaued at levels of 20–35 mg/L as the natural-gas well matured (150 to >450 days, Figure 2D). This period of intermediate sulfate and sulfide concentrations was associated with a higher abundance of 16S rRNA gene sequences for *Arcobacter* (Figure 2A) and could be related to the reoxidation of reduced sulfur species during this time. Also present in the *Arcobacter* genomes was the *sqr* gene responsible for oxidation of sulfide to polysulfides, the *pshABC* gene which reduces sulfur and thiosulfate to sulfide, and the *cysIJ* gene which oxidizes sulfide to sulfite (Kanehisa and Goto, 2000). Both isolates are also capable of reducing tetrathionate to thiosulfate (*ttr* gene) (Kanehisa and Goto, 2000). One isolate, *Arcobacter sp.* MARC-MIP3H16, contains the gene responsible for oxidation of thiosulfate to sulfite (*sseA*) (Kanehisa and Goto, 2000).

We detected 36 cytochromes in the genome of *Marinobacter sp.* UTICA-S1B6, some of which may be involved in extracellular electron transfer to redox active species (e.g., iron and oxygen). One of these cytochromes was a (per)oxidase also detected in *Arcobacter sp.* MARC-MIP3H16. Additionally, *Marinobacter* contained a thiol (per)oxidase and a catalase (per)oxidase. Closely related *Marinobacter subterrani* is known to oxidize Fe(II), and *Marinobacter aquaeolei* oxidizes Fe(II) from metal structures during growth in biofilms (Singer et al., 2011; Bonis and Gralnick, 2015). The (per)oxidase cytochrome is one of many cytochromes thought to be responsible for iron oxidation, while a type II secretion system is associated with *Marinobacter aquaeolei’s* ability to form a biofilm that interacts with metal surfaces (Singer et al., 2011). Indeed, the (per)oxidase encoding gene (pfam 00141) in the genome of *Marinobacter* sp. UTICA-S1B6 is homologous to the peroxidase encoding gene of *Marinobacter subterrani* JG233 (82% identity, 1234 bit score) and *Marinobacter aquaeolei* VT8 (88% identity, bit score 1321). Interestingly, *Marinobacter* sp. UTICA-S1B6 also contains the type II secretion system, suggesting a capacity to attach to reduced iron surfaces (e.g., minerals such as clays, silicates, and carbonates common in shale, carbon steel casings) and oxidize reduced forms of iron abundant within the organic-rich subsurface systems responsible for the generation of petroleum (i.e., source rocks such as shale, mudstones, organic-rich limestones, etc.) (Singer et al., 2011).

### *Marinobacter* and *Arcobacter* Possess Unique Virulence Strategies in Produced Fluid

During our assessment of these *Marinobacter* and *Arcobacter* genomes, we identified genes comprising a type VI secretion system (Figures 5A,B). This system is best described as a phage-like apparatus which can be used to lyse other cells by injecting enzymes designed to degrade cellular components. Although energetically costly (Alteri and Mobley, 2016), this virulence strategy may provide a competitive advantage to these taxa under nutrient limited conditions or environmental stress (Chakraborty et al., 2011; Alteri and Mobley, 2016). Said strategy could also contribute to horizontal gene transfer within this system (Borgeaud et al., 2015). To date, no literature has been published identifying this virulence survival mechanism in the environment that occurs following hydraulic fracturing, although there is strong genomic evidence for persistent viral attacks to these taxa (Daly et al., 2016). Indeed, the genomes of *M.* UTICA-S1B6, *A.* UTICA-S4D1, and *A.* MARC-MIP3H16 all contain genes indicative of a type I CRISPR-Cas system which serve as acquired immunity to predatory bacteriophage (Makarova et al., 2015).

### Isolate Genomes Largely Agree With Metagenomic Data

The key pathways summarized above for the isolate genome of *Marinobacter* sp. UTICA-S1B6 were also discovered with high homology in the *Marinobacter* metagenome sampled from fluids produced on day 34 from Marcellus Shale natural-gas well Marcellus-4 (bit score >200, identity >45.9%). One notable exception was the difference in the pathway for aromatic hydrocarbon degradation. The metagenome contained genes associated with catechol degradation via *ortho*-cleavage as opposed to the isolate genome which contained genes encoding for the *meta*-cleavage pathway (Kanehisa and Goto, 2000; Caspi et al., 2014). Several studies report that the *meta-*pathway is used for both non-substituted aromatics in addition to alkyl and halo- substituted aromatics such as toluene and chlorobenzene, whereas the *ortho*-pathway is generally observed exclusively during growth on non-substituted benzene or phenol (Mars et al., 1997; Veenagayathri and Vasudevan, 2011). The exclusive possession of the *ortho*-pathway by *Marinobacter* in the produced fluid metagenome may indicate sole preference for non-substituted or hydroxylated aromatic hydrocarbons in the environment. In addition, the gene for utilizing nitroalkanes through conversion to an aldehyde or ketone plus nitrate was discovered in the metagenomes with high homology to *Marinobacter*, but was absent in the genomes.

Largely, genes belonging to *Arcobacter* mined from the metagenome of the Marcellus Shale natural-gas well Marcellus-4 at days 34 and 93 meeting homology cutoffs agreed with the isolate genomes. However, a few exceptions are noted (Supplementary Table S1) including the absence of two sulfur genes in the metagenomes, *cysJ* (sulfide to sulfite) and *sseA* (thiosulfate to sulfite), as well as *TMO* (transforms trimethylamine n-oxide to trimethylamine), which were all present in the *Arcobacter* isolate genome.

## Discussion

### *Marinobacter* and *Arcobacter* Dominate Early Produced Fluid Communities Through Unique Osmoadaptations and Defense Mechanisms

*Marinobacter* are one of a dozen microbial taxa commonly found in producing natural-gas wells from hydraulically stimulated formations across the United States. (Mouser et al., 2016). The presence of this taxa during early production in Marcellus-4 is consistent with trends reported for other geographically and lithologically distinct unconventional hydrocarbon-producing systems (e.g., shales, mudstones, organic-rich limestones), which show *Marinobacter* present in early flowback fluid samples (<49 days after flowback began) but largely absent after that time (>49 days) (Davis et al., 2012; Fichter et al., 2012; Struchtemeyer and Elshahed, 2012; Mohan et al., 2013; Cluff et al., 2014). Similarly, the temporal trends reported here for *Arcobacter* abundance (Figure 2A) are consistent with those reported by Wuchter et al. (2013), who detected *Arcobacter marinus* in produced fluids from the Antrim Shale ranging from 35 to 85% of the total ε-proteobacteria abundance during the 5 months after hydraulic fracturing. Previous studies on fluids produced from the Marcellus Shale report *Arcobacter* abundance peaking (upwards of 66% of total reported microbial community based on the 16S rRNA gene) within the first 2 weeks after production begins and decreasing thereafter (Mohan et al., 2013; Cluff et al., 2014). This consistent detection of *Marinobacter* during the first few weeks after fracturing and *Arcobacter* during the first few months after fracturing suggests these taxa play an important, albeit fleeting, role in the subsurface following natural-gas well completion and hydraulic fracturing.

Based on genomic and metagenomic inference, there are several survival strategies these organisms possess that could enable their persistence in fluids derived from tight shales and other unconventional hydrocarbon-producing systems during this transitional biogeochemical phase. Arguably, the largest bottleneck for the survival of microorganisms in an ecosystem such as those generated following fracturing of shales or other unconventional systems is the high salinity observed in produced fluids from these formations, which is known to increase as hydrocarbon production proceeds. Both *Arcobacter* and *Marinobacter* possess strategies for adapting to high salt, namely via anti-porters using a “salt-in” strategy, where cells import ions into the cytoplasm to avoid osmotic stress under increasing concentrations in the environment. Both taxa can also synthesize the osmolyte ectoine and import extracellular osmolytes. Additionally, *Marinobacter* can produce osmoprotectant glycine betaine in response to high salinity, which can subsequently be fermented by other microbial community members to fuel methanogenesis (Daly et al., 2016).

The ability to prey upon other bacterial taxa is a survival strategy that could allow predatorial taxa to have a significant advantage in subsurface systems with strong competition for nutrient or electron acceptor resources. The type VI secretion system detected in both *Marinobacter* and *Arcobacter* genomes may allow these taxa to attack neighboring cells with a phage-like appendage, insert effector proteins, and capture cellular materials from the victim including lipids, polysaccharides, proteins, amino acids, and nucleic acids. Besides antagonism, the type VI secretion system may be used for cell signaling, biofilm remodeling, or lysing phage-infected bacteria (Russell et al., 2014). Interestingly, *Marinobacter hydrocarbonoclasticus* grown on hexadecane overexpressed type VI secretion system proteins relative to controls. The authors concluded a role for the type VI secretion system in alkane assimilation (Vaysse et al., 2009). As both *Arcobacter* and *Marinobacter* possess this system, its presence could confer their advantage in fluids derived from tight shales and other unconventional hydrocarbon-producing systems through biofilm formation, virulence, or hydrocarbon assimilation. Furthermore, the presence of CRISPR-associated encoding genes in the genomes of all three isolates may confer additional immunity in this system in the presence of lysogenic phage (Daly et al., 2016).

### *Marinobacter* and *Arcobacter* Are Carbon Opportunists

Our investigation into the metabolic potential of *Marinobacter* sp. UTICA-S1B6 revealed the capacity for complex carbon oxidation, including aliphatic compounds, aromatic compounds, and haloacids. Our phylogenetic analysis based on the 16S rRNA gene showed *M.* UTICA-S1B6 is closely related to *Marinobacter hydrocarbonoclasticus* (96.6%), a bacterium isolated from seawater collected near an oil refinery in the Mediterranean Sea (Mounier et al., 2014). *M. hydrocarbonoclasticus* is able to degrade a wide variety of aliphatic and aromatic hydrocarbons through oleolytic biofilm formation. *Marinobacter* sp. UTICA-S1B6 could access and transform hydrocarbons in a similar manner based on our genomic evaluation by coupling these reactions to oxygen and/or nitrate reduction, enabling its persistence during the first few weeks of flowback before oxygenated resources are depleted. Furthermore, *Marinobacter* may persist as labile carbon substrates are depleted due to its ability to utilize a wide range of organic carbon sources much like other opportunistic *Marinobacter* species (Singer et al., 2011).

An autotrophic metabolism potentially confers advantage for *Arcobacter* species among a primarily heterotrophic and fermentative microbial community. As the natural-gas well matures and the sources of injected labile carbon dwindle, *Arcobacter* may alternate between heterotrophic and autotrophic metabolisms. Further experimentation is required to confirm its ability to fix CO_2_, but geochemical and genomic evidence suggest this enzymatic pathway may be important for the persistence of *Arcobacter* during the first few months following hydraulic fracturing and hydrocarbon production within a given natural-gas well. Much like opportunistic *Marinobacter*, *Arcobacter* species in unconventional systems may also utilize diverse carbon substrates (organic and inorganic), electron acceptors (oxygen, nitrate), and electron donors (reduced sulfur and iron species) that are generally present during early flowback.

### Importance of *Marinobacter* and *Arcobacter* on Biogeochemical Cycling in Flowback and Produced Fluids

Nitrate is added in certain oil and gas systems to thermodynamically control growth of sulfate reducers and fermenters, thereby reducing biocorrosion damage to infrastructure through sulfide and/or acid generation (Voordouw, 2008; Arensdorf et al., 2009; Voordouw et al., 2009; An et al., 2017). As a result, there is need to understand the utilization of both organic and inorganic nitrogen species by microorganisms in subsurface environments such as shale or other unconventional hydrocarbon-bearing systems. In contrast to other subsurface terrestrial systems like deep groundwater environments that have limited nitrogen sources (Kutvonen et al., 2015; Bomberg and Ahonen, 2017), excess sources of fixed nitrogen are available in fluids produced from shale or other unconventional hydrocarbon resources.

Our results show that both TDN and ammonia increase significantly as production proceeds in six hydraulically fractured natural-gas wells in the northern Appalachian Basin. The individual constituents of TDN may originate from a variety of anthropogenic and indigenous sources. Since nitrate was below detection in produced fluids, it is not a considerable source of TDN here. In the case of the (Utica-3, Utica-6, Utica-7, and Utica-8) natural-gas wells, produced fluids from other hydraulically fractured natural-gas wells were used as a source of slickwater for hydraulic fracturing (i.e., recycled produced fluid), which may partially explain high initial TDN values. In terms of other nitrogen sources, organic nitrogen is frequently added to fracture fluid formulations for several different purposes, including clay stabilization, scale inhibition, friction reduction, gel formation, and as surfactants and solvents (Elsner and Hoelzer, 2016). For example, urea was one chemical component of the hydraulic fracturing fluid in the Marcellus Shale natural-gas well Marcellus-4, and its enzymatic conversion to ammonia and carbon dioxide may contribute to TDN and ammonia trends in these wells. Ammonia is also a potential byproduct of (poly)acrylamide and related polymer degradation (biotic or abiotic). Polyacrylamides are contained in many slickwater fracturing fluid recipes (Elsner and Hoelzer, 2016), and are listed on the FracFocus report for the three Marcellus and Utica-Point Pleasant natural-gas wells where these three strains were isolated (*Marinobacter* sp. UTICA-S1B6, *Arcobacter* sp. S4D1, and *Arcobacter sp.* MARC-MIP3H16) (Supplementary Figures S2–S4). Interestingly, *Marinobacter* contains amidase-encoding genes, suggesting that this taxa could degrade amide-based polymers in this system, such as polyacrylamides. Furthermore, both *Marinobacter* and *Arcobacter* can produce nitrate from nitroalkanes, a pre-cursor biochemical process that could enable denitrification or dissimilatory nitrate reduction despite low (<1 ppm) nitrate concentrations in produced fluids.

Nitrogen fixation is energetically costly, and the presence of bioavailable nitrogen sources (e.g., nitrate, urea, amines, ammonia) makes it unlikely nitrogen fixation occurs in these unconventional hydrocarbon-producing systems (Houlton et al., 2008). Fixed sources of nitrogen must therefore be biologically produced or sourced within the rock (e.g., shale). *Arcobacter* may produce ammonia during early production through dissimilatory nitrate reduction. A methanogen that thrives during later production, *Methanohalophilus*, may also contribute to ammonia production during methanogenesis fueled by methylamines (Borton et al., 2018). Altogether, it is plausible that *Marinobacter* and *Arcobacter* in early production of hydraulically fractured natural-gas wells contribute to marked increases in total nitrogen concentrations and ammonia in produced fluids by reducing nitrate and/or degrading xenobiotic organic nitrogen sources injected by well operators.

The primary cause of natural-gas well infrastructure fouling is attributed to the acidic and reactive properties of sulfides, making the sulfur cycle a vitally important process to understand in this system (Pirzadeh et al., 2014; Booker et al., 2017). Previous studies indicate thiosulfate reduction by *Halanaerobium spps.* as the primary mechanism for sulfide formation in later produced fluids (Liang et al., 2016; Booker et al., 2017). Contributing to this biogeochemical cycle, *Arcobacter* spp. have the capacity to refuel sulfide production, at least temporarily, through oxidation of reduced sulfur species (e.g., sulfides, sulfur) to thiosulfate and sulfate. This process would initially reduce sulfide levels until oxygen and/or nitrate are depleted from the system and provide electron acceptors (e.g., thiosulfate, sulfate) for other dominant taxa. Although *Arcobacter* spp. studied here have the ability to oxidize sulfide to sulfate, we propose thiosulfate to be the primary endpoint of *Arcobacter* sulfur metabolism due to (1) the lack of sulfate present in later produced fluids, and (2) the presence of known thiosulfate reducers in these wells (e.g. *Halanaerobium*).

## Conclusion

Bacterial taxa common to early production stages of hydraulically fractured natural-gas wells play significant roles in carbon, nitrogen, and sulfur cycling in flowback and produced fluids. *Marinobacter* can oxidize hydrocarbons as well as capture carbon, nitrate, and sulfur species from alkane-derived hydrocarbons, which are most likely present from fracture fluid additives and/or brines produced from unconventional hydrocarbon systems during the early stages of natural-gas production. *Arcobacter* uniquely participates in carbon and sulfur cycling through coupling chemosynthesis to oxidation of reduced sulfur species, which may fuel heterotrophic, fermentative, and/or thiosulfate-reducing microorganisms. These taxa may further contribute to high ammonia concentrations in these natural-gas wells through urea conversion or nitrate reduction. Our genomic and experimental investigations of *Marinobacter* and *Arcobacter* physiology reveals the impact these slight to moderate halophilic taxa have on biogeochemical cycles during early production stages in hydraulic fractured natural-gas wells as oxygen and carbon resources are diminishing and salinities increase.

## Significance

This work contributes to our understanding of carbon, nitrogen, and sulfur microbial metabolisms in saline produced fluids from fractured natural-gas wells.

## Author Contributions

PM and JP conceived and designed the experiments with assistance from MW and KW. ME wrote the manuscript. JP performed the laboratory-based experiments. JP, SW, and JS acquired the SEM images. NN compiled geochemical data and contributed a figure. JP and ME performed the genomic analyses. DC, MW, KW, and PM designed field experiments while AH, RD, SW, and JS carried out field sampling and performed the geochemical measurements. KW and RD extracted DNA and processed metagenomes. ME performed the metagenomic analyses and assisted with geochemical measurements. TD performed the CO_2_ analysis.

## Conflict of Interest Statement

The authors declare that the research was conducted in the absence of any commercial or financial relationships that could be construed as a potential conflict of interest.
