# Supplementary material for: Members of Marinobacter and Arcobacter Influence System Biogeochemistry During Early Production of Hydraulically Fractured Natural Gas Wells in the Appalachian Basin
Source: Front Microbiol. 2018 Nov 15;9:2646. doi: 10.3389/fmicb.2018.02646 (PMC6249378; doi:10.3389/fmicb.2018.02646)
Supplement: Supplementary file 2 [file Data_Sheet_2.PDF]

frontiers

Supplementary Material

Influence of Marinobacter and Arcobacter taxa on system biogeochemistry during early production of hydraulically fractured shale gas wells in the Appalachian Basin

Morgan V. Evans [1], Jenny Panescu[1], Andrea J. Hanson[2], Julie Sheets[3], Susan A. Welch [3], Nicholas Nastasi[1], Rebecca A. Daly [4], David R. Cole [3], Thomas H. Darrah [3], Michael J. Wilkins [3], Kelly C. Wrighton[4], Paula J. Mouser [1,5]

*** Correspondence:** Paula Mouser: Paula.Mouser@unh.edu

Supplementary Data:

Table S1 (uploaded separately as an excel file): Pulled metagenome genes from MIP-3H with bit-score and identity % scores

Table S2: *Marinobacter* and *Arcobacter* salinity growth curve results

Figure S1: Biolog Plate Reader Results

Figure S2-S4: FracFocus Reports from Utica S1, S4, and Marcellus MIP-3H

Marinobacter Aerobic Growth & Rates		
Salinity (% NaCl)	average k	StdDev
1	0.032	0.005
2.5	0.071	0.031
5	0.061	0.009
7.5	0.044	0.005
10	0.078	0.008
12.5	0.081	0.006
15	0.061	0.003
20	0.018	0.003
Marinobacter Anaerobic Growth & Rates		
Salinity (% NaCl)	average k	StdDev
0.875	0.052	0.005
2.5	0.084	0.003
5	0.090	0.006
7.5	0.045	0.003
10	0.025	0.003
12.5	0.024	0.003
Arcobacter Aerobic Growth & Rates		
Salinity (% NaCl)	average k	StdDev
0.5	0.004	0.003
2	0.110	0.022
4	0.583	0.020
6	0.230	0.017
8	0.107	0.002
10	0.102	0.027
12	0.103	0.004
14	0.002	0.012

Table S2. *Marinobacter* and *Arcobacter* salinity curve results

PM1 Carbon Sources

	1	2	3	4	5	6	7	8	9	10	11	12	
A	Negative Control	L-Arabinose	N-Acetyl-D-Glucosamine	D-Saccharic Acid	Succinic Acid	D-Galactose	L-Aspartic Acid	L-Proline	D-Alanine	D-Trehalose	D-Mannose	Dulcitol	
	**0.014**	**0.049**	**0.00575**	**0.03125**	**0.21675**	**0.03175**	**0.1195**	**0.1365**	**≤ 0**	**0.02575**	**0.023**	**0.0285**	Arc. MIP3H16
	**0.02275**	**0.0155**	**0.01475**	**0.03875**	**0.1365**	**0.02775**	**0.02525**	**0.2135**	**0.02125**	**0.03125**	**0.0255**	**0.04975**	Mar. S1B6
B	D-Serine	D-Sorbitol	Glycerol	L-Fucose	D-Glucuronic Acid	D-Gluconic Acid	D,L-α-Glycerol-Phosphate	D-Xylose	L-Lactic Acid	Formic Acid	D-Mannitol	L-Glutamic Acid	
	**0.025**	**0.026**	**0.03225**	**0.0185**	**0.024**	**0.04725**	**0.02725**	**0.054**	**0.15275**	**0.0135**	**≤ 0**	**0.1275**	Arc. MIP3H16
	**0.0155**	**0.02975**	**0.02475**	**0.0215**	**0.03125**	**0.03025**	**0.0265**	**0.01275**	**0.15825**	**0.01525**	**≤ 0**	**0.1625**	Mar. S1B6
C	D-Glucose-6-Phosphate	D-Galactonic Acid-γ-Lactone	D,L-Malic Acid	D-Ribose	Tween 20	L-Rhamnose	D-Fructose	Acetic Acid	α-D-Glucose	Maltose	D-Melibiose	Thymidine	
	**0.0165**	**0.0085**	**0.137**	**0.01425**	**0.01725**	**0.032**	**0.056**	**0.05125**	**0.037**	**0.0325**	**0.0135**	**0.02575**	Arc. MIP3H16
	**0.0365**	**0.0195**	**0.15825**	**≤ 0**	**0.08825**	**0.035**	**0.032**	**0.173**	**0.0335**	**0.03125**	**0.0165**	**0.0385**	Mar. S1B6
D	L-Asparagine	D-Aspartic Acid	D-Glucosaminic Acid	1,2-Propanediol	Tween 40	α-Keto-Glutaric Acid	α-Keto-Butyric Acid	α-Methyl-D-Galactoside	α-D-Lactose	Lactulose	Sucrose	Uridine	
	**0.05675**	**0.002**	**0.025**	**0.02875**	**0.0135**	**0.09475**	**0.03**	**0.041**	**0.0285**	**0.0095**	**0.02275**	**0.04725**	Arc. MIP3H16
	**0.03925**	**0.01125**	**0.0225**	**0.02725**	**0.05075**	**0.09**	**0.06375**	**0.03475**	**0.026**	**0.01475**	**0.02125**	**0.038**	Mar. S1B6
E	L-Glutamine	m-Tartaric Acid	D-Glucose-1-Phosphate	D-Fructose-6-Phosphate	Tween 80	α-Hydroxy Glutaric Acid-γ-Lactone	α-Hydroxy Butyric Acid	β-Methyl-D-Glucoside	Adonitol	Maltotriose	2-Deoxy Adenosine	Adenosine	
	**0.195**	**0.00425**	**0.024**	**0.057**	**0.02275**	**0.062**	**0.01525**	**0.03475**	**0.04325**	**0.032**	**0.01925**	**0.0185**	Arc. MIP3H16
	**0.0295**	**0.0295**	**0.01**	**≤ 0**	**0.0805**	**0.02775**	**0.053**	**0.028**	**0.03175**	**0.02725**	**0.019**	**0.01**	Mar. S1B6
F	Glycyl-L-Aspartic Acid	Citric Acid	m-Inositol	D-Threonine	Fumaric Acid	Bromo Succinic Acid	Propionic Acid	Mucic Acid	Glycolic Acid	Glyoxylic Acid	D-Cellobiose	Inosine	
	**0.027**	**0.062**	**0.04125**	**0.0325**	**0.098**	**0.07575**	**0.1**	**0.05175**	**0.027**	**0.0035**	**0.038**	**≤ 0**	Arc. MIP3H16
	**0.022**	**0.2395**	**0.02625**	**0.01375**	**0.1005**	**≤ 0**	**0.1425**	**0.0285**	**0.01525**	**0.00025**	**0.03925**	**≤ 0**	Mar. S1B6
G	Glycyl-L-Glutamic Acid	Tricarballylic Acid	L-Serine	L-Threonine	L-Alanine	L-Alanyl-Glycine	Acetoacetic Acid	N-Acetyl-α-D-Mannosamine	Mono Methyl Succinate	Methyl Pyruvate	D-Malic Acid	L-Malic Acid	
	**0.034**	**0.103**	**0.042**	**0.01975**	**≤ 0**	**0.0155**	**0.051**	**0.0575**	**0.18025**	**0.008**	**≤ 0**	**0.325**	Arc. MIP3H16
	**0.02675**	**0.03675**	**0.01375**	**0.019**	**0.02375**	**0.02525**	**0.049**	**0.035**	**0.03825**	**≤ 0**	**0.04975**	**0.29**	Mar. S1B6
H	Glycyl-L-Proline	p-Hydroxy Phenyl Acetic Acid	m-Hydroxy Phenyl Acetic Acid	Tyramine	D-Psicose	L-Lyxose	Glucuronamide	Pyruvic Acid	L-Galactonic Acid-γ-Lactone	D-Galacturonic Acid	Phenylethyl-amine	2-Aminoethanol	
	**0.0105**	**0.0605**	**0.004**	**0.0165**	**0.038**	**≤ 0**	**0.0065**	**0.216**	**0.00975**	**0.0175**	**≤ 0**	**0.015**	Arc. MIP3H16
	**0.03675**	**0.0035**	**0.0155**	**0.03125**	**0.02325**	**≤ 0**	**≤ 0**	**0.11425**	**0.0185**	**0.027**	**≤ 0**	**0.0265**	Mar. S1B6

Fig. S1) Each isolate was analyzed in duplicate Biolog Phenotypic Microarray Plate 1 (PM1), a 96-well plate containing distinct carbon sources in each well. The plates were inoculated aerobically for 72 hours at 30°C in an automated spectrophotometer which collected bihourly absorbance readings at 600 nm. The results shown indicate the average increase in absorbance values of the duplicate inoculated plates over the duplicate uninoculated plates (negative control) over the period of incubation.

Hydraulic Fracturing Fluid Product Component Information Disclosure

Job Start Date:	
Job End Date:	
State:	West Virginia
County:	
API Number:	
Operator Name:	
Well Name and Number:	
Longitude:	
Latitude:	
Datum:	
Federal/Tribal Well:	
True Vertical Depth:	7,483
Total Base Water Volume (gal):	10,647,966
Total Base Non Water Volume:	0

Hydraulic Fracturing Fluid Composition:

Trade Name	Supplier	Purpose	Ingredients	Chemical Abstract Service Number (CAS #)	Maximum Ingredient Concentration in Additive (% by mass)**	Maximum Ingredient Concentration in HF Fluid (% by mass)**	Comments
Ingredients shown above are subject to 29 CFR 1910.1200(i) and appear on Material Safety Data Sheets (MSDS). Ingredients shown below are Non-MSDS.							
Proppant Transport	Schlumberger	Corrosion Inhibitor, Scale Inhibitor, Biocide, AntiFoam Agent, Acid, Breaker, Gelling Agent, Friction Reducer, Iron Control Agent, Fluid Loss Additive					
			Water (Including Mix Water Supplied by Client)*	NA		87.63568	
			Quartz, Crystalline silica	14808-60-7	99.06784	12.21724	
			Hydrochloric acid	7647-01-0	0.66726	0.08228	
			Ammonium sulfate	7783-20-2	0.06845	0.00844	
			Guar gum	9000-30-0	0.05865	0.00724	
			Acrylamide, 2-acrylamido-2-methylpropanesulfonic acid, sodium salt polymer	38193-60-1	0.05052	0.00623	
			Glutaraldehyde	111-30-8	0.02831	0.00349	
			Ethanol, 2,2',2''-nitrilotris-, 1,1',1''-tris(dihydrogen phosphate), sodium salt	68171-29-9	0.00971	0.00120	
			Diammonium peroxidisulphate	7727-54-0	0.00601	0.00074	

			Polymer of 2-acrylamido-2-methylpropanesulfonic acid sodium salt and methyl acrylate	136793-29-8	0.00541	0.00067	
			Alkyl(c12-16) dimethylbenzyl ammonium chloride	68424-85-1	0.00506	0.00062	
			Sodium erythorbate	6381-77-7	0.00436	0.00054	
			Trisodium ortho phosphate	7601-54-9	0.00427	0.00053	
			Urea	57-13-6	0.00332	0.00041	
			Polypropylene glycol	25322-69-4	0.00294	0.00036	
			Methanol	67-56-1	0.00252	0.00031	
			Fatty acids, tall-oil	61790-12-3	0.00156	0.00019	
			Thiourea, polymer with formaldehyde and 1-phenylethanone	68527-49-1	0.00129	0.00016	
			Ethylene Glycol	107-21-1	0.00121	0.00015	
			Non-crystalline silica (impurity)	7631-86-9	0.00084	0.00010	
			Vinylidene chloride/methylacrylate copolymer	25038-72-6	0.00080	0.00010	
			Sodium sulfate	7757-82-6	0.00078	0.00010	
			Alcohols, C14-15, ethoxylated (7EO)	68951-67-7	0.00061	0.00008	
			Ethanol	64-17-5	0.00061	0.00007	
			Propargyl alcohol	107-19-7	0.00041	0.00005	
			2-Propenamid (impurity)	79-06-1	0.00017	0.00002	
			Hexadec-1-ene	629-73-2	0.00014	0.00002	
			1-Octadecene (C18)	112-88-9	0.00007	0.00001	
			Dimethyl siloxanes and silicones	63148-62-9	0.00005	0.00001	
			Tetrasodium ethylenediaminetetraacetate	64-02-8	0.00009	0.00001	
			Dodecamethylcyclohexasiloxane	540-97-6			
			Siloxanes and silicones, dimethyl, reaction products with silica	67762-90-7	0.00001		
			Octamethylcyclotetrasiloxane	556-67-2			
			poly(tetrafluoroethylene)	9002-84-0	0.00001		
			Formaldehyde	50-00-0	0.00001		
			Copper(II) sulfate	7758-98-7			
			Decamethyl cyclopentasiloxane	541-02-6			
			Magnesium silicate hydrate (talc)	14807-96-6	0.00002		
FR Pro 150	ECM	Friction Reduction					
			Water	7732-18-5	50.00000	0.01575	
			Polyacrylamide-co-acrylic acid	9003-06-9	32.00000	0.01008	
			Sodium Chloride	7647-14-5	15.00000	0.00472	
			Alcohol Ethoxylate Surfactants	Trade	5.00000	0.00157	
			Petroleum Distillate	64742-47-8	25.00000		

* Total Water Volume sources may include fresh water, produced water, and/or recycled water
** Information is based on the maximum potential for concentration and thus the total may be over 100%

Note: For Field Development Products (products that begin with FDP), MSDS level only information has been provided.
Ingredient information for chemicals subject to 29 CFR 1910.1200(i) and Appendix D are obtained from suppliers Material Safety Data Sheets (MSDS)

Hydraulic Fracturing Fluid Product Component Information Disclosure

Job Start Date:	
Job End Date:	
State:	Ohio
County:	
API Number:	
Operator Name:	
Well Name and Number:	
Longitude:	
Latitude:	
Datum:	
Federal/Tribal Well:	
True Vertical Depth:	9,647
Total Base Water Volume (gal):	7,556,598
Total Base Non Water Volume:	0

Hydraulic Fracturing Fluid Composition:

Trade Name	Supplier	Purpose	Ingredients	Chemical Abstract Service Number (CAS #)	Maximum Ingredient Concentration in Additive (% by mass)**	Maximum Ingredient Concentration in HF Fluid (% by mass)**	Comments
Fresh water	Stingray	Carrier					
			Water	7732-18-5	100.00000	88.41106	
40/70 White	Steubenville/Cadiz	Proppant					
			Sand	14808-60-7	100.00000	10.83323	
Muriatic Acid	Axiall, LLC	Acid					
			Water	7732-18-5	60.00000	0.19207	
			Hydrogen chloride	7647-01-0	40.00000	0.12804	
100 mesh	Minerva/Cadiz	Proppant					
			Sand	14808-60-7	100.00000	0.22916	
FRA 409	Weatherford	Friction Reducer					
			Ethanaminium, N,N,N-trimethyl-2-[(1-oxo-2-propenyl)oxy]-, chloride, polymer with 2-propenamide	69418-26-4	70.00000	0.08128	
			Proprietary	Proprietary	30.00000	0.03483	
			Petroleum Distillate	64742-47-8	10.00000	0.01161	
			Alcohols, C12-14-secondary, ethoxylated	84133-50-6	5.00000	0.00581	
			Adipic acid	124-04-9	3.00000	0.00348	
B-84	X-Chem, LLC	Biocide					
			Water	7732-18-5	55.50000	0.01753	

			Glutaraldehyde	111-30-8	27.00000	0.00853		
			Didecyl dimethyl ammonium chloride	7173-51-5	8.00000	0.00253		
			n-Alkyl dimethyl benzyl ammonium chloride	68424-85-1	5.50000	0.00174		
			Ethanol	64-17-5	4.00000	0.00126		
VBL-29	X-Chem, LLC	Breaker						
			Water	7732-18-5	90.00000	0.02674		
			Hydrogen Peroxide	7722-84-1	10.00000	0.00297		
Plexgel 907L-EB	Chemplex SOLVAY	Viscosifier						
			Guar Gum	9000-30-0	50.00000	0.00775		
			Distillate(petroleum), hydrotreated light	64742-47-8	50.00000	0.00775		
			Organophylic Clay	Proprietary	2.00000	0.00031		
			Alcohol ethoxylate	34398-01-1	0.99000	0.00015		
			Cyrstalline Silica	14808-60-7	0.06000	0.00001		
SC-30	X-Chem, LLC	Scale Inhibitor						
			Water	7732-18-5	70.00000	0.00746		
			Sodium Polyacrylate	Proprietary	30.00000	0.00320		
TCA 6038F	X-Chem, LLC	Corossion Inhibitor						
			Water	7732-18-5	80.00000	0.00229		
			Methanol	67-56-1	20.00000	0.00057		
Ingredients shown above are subject to 29 CFR 1910.1200(i) and appear on Material Safety Data Sheets (MSDS). Ingredients shown below are Non-MSDS.								

* Total Water Volume sources may include fresh water, produced water, and/or recycled water
** Information is based on the maximum potential for concentration and thus the total may be over 100%

Note: For Field Development Products (products that begin with FDP), MSDS level only information has been provided.
Ingredient information for chemicals subject to 29 CFR 1910.1200(i) and Appendix D are obtained from suppliers Material Safety Data Sheets (MSDS)

Hydraulic Fracturing Fluid Product Component Information Disclosure

Job Start Date:	
Job End Date:	
State:	Ohio
County:	
API Number:	
Operator Name:	
Well Name and Number:	
Longitude:	
Latitude:	
Datum:	
Federal/Tribal Well:	
True Vertical Depth:	9,633
Total Base Water Volume (gal):	7,691,796
Total Base Non Water Volume:	0

Hydraulic Fracturing Fluid Composition:

Trade Name	Supplier	Purpose	Ingredients	Chemical Abstract Service Number (CAS #)	Maximum Ingredient Concentration in Additive (% by mass)**	Maximum Ingredient Concentration in HF Fluid (% by mass)**	Comments
Fresh water	Stingray	Carrier					
			Water	7732-18-5	100.00000	87.06859	
40/70 White	Steubenville/Cadiz	Proppant					
			Sand	14808-60-7	100.00000	12.18420	
Muriatic Acid	Axiall, LLC	Acid					
			Water	7732-18-5	60.00000	0.19202	
			Hydrogen chloride	7647-01-0	40.00000	0.12801	
100 mesh	Minerva/Cadiz	Proppant					
			Sand	14808-60-7	100.00000	0.22419	
FRA 409	Weatherford	Friction Reducer					
			Ethanaminium, N,N,N-trimethyl-2-[(1-oxo-2-propenyl)oxy]-, chloride, polymer with 2-propenamide	69418-26-4	70.00000	0.07907	
			Proprietary	Proprietary	30.00000	0.03389	
			Petroleum Distillate	64742-47-8	10.00000	0.01130	
			Alcohols, C12-14-secondary, ethoxylated	84133-50-6	5.00000	0.00565	
			Adipic acid	124-04-9	3.00000	0.00339	
B-84	X-Chem, LLC	Biocide					
			Water	7732-18-5	55.50000	0.01759	

			Glutaraldehyde	111-30-8	27.00000	0.00856	
			Didecyl dimethyl ammonium chloride	7173-51-5	8.00000	0.00254	
			n-Alkyl dimethyl benzyl ammonium chloride	68424-85-1	5.50000	0.00174	
			Ethanol	64-17-5	4.00000	0.00127	
VBL-29	X-Chem, LLC	Breaker					
			Water	7732-18-5	90.00000	0.02801	
			Hydrogen Peroxide	7722-84-1	10.00000	0.00311	
Plexgel 907L-EB	Chemplex SOLVAY	Viscosifier					
			Distillate(petroleum), hydrotreated light	64742-47-8	50.00000	0.00709	
			Guar Gum	9000-30-0	50.00000	0.00709	
			Organophylic Clay	Proprietary	2.00000	0.00028	
			Alcohol ethoxylate	34398-01-1	0.99000	0.00014	
			Cyrstalline Silica	14808-60-7	0.06000	0.00001	
SC-30	X-Chem, LLC	Scale Inhibitor					
			Water	7732-18-5	70.00000	0.00742	
			Sodium Polyacrylate	Proprietary	30.00000	0.00318	
TCA 6038F	X-Chem, LLC	Corossion Inhibitor					
			Water	7732-18-5	80.00000	0.00194	
			Methanol	67-56-1	20.00000	0.00049	
Ingredients shown above are subject to 29 CFR 1910.1200(i) and appear on Material Safety Data Sheets (MSDS). Ingredients shown below are Non-MSDS.							

* Total Water Volume sources may include fresh water, produced water, and/or recycled water
** Information is based on the maximum potential for concentration and thus the total may be over 100%

Note: For Field Development Products (products that begin with FDP), MSDS level only information has been provided.
Ingredient information for chemicals subject to 29 CFR 1910.1200(i) and Appendix D are obtained from suppliers Material Safety Data Sheets (MSDS)
